# Learning from One-Health approaches to explore links between farming practices, animal, human and ecosystem health in Nigeria

**DOI:** 10.3389/fnut.2024.1216484

**Published:** 2024-01-26

**Authors:** Akaninyene Otu, Obiageli Onwusaka, Clement Meseko, Emmanuel Effa, Bassey Ebenso, Isong Isong Abraham, Jeremiah Ijomanta, Ayokunle Omileye, Chinenye Emelife, Sunday Eziechina, Kabiru Suleiman, Chinwe Ochu, Victor Adetimirin

**Affiliations:** ^1^Department of Research and Innovation, Foundation for Healthcare Innovation and Development (FHIND), Calabar, Cross River, Nigeria; ^2^Department of Internal Medicine, College of Medical Sciences, University of Calabar, Calabar, Cross River, Nigeria; ^3^Department of Public Health, Faculty of Allied Medical Sciences, College of Medical Sciences, University of Calabar, Calabar, Nigeria; ^4^Regional Laboratory for Animal Influenza and Transboundary Animal Diseases (TADs), National Veterinary Research Institute, Vom, Plateau, Nigeria; ^5^Department of Internal Medicine, Edward Francis Small Teaching Hospital, Banjul, Gambia; ^6^Nuffield Centre for International Health and Development, Leeds Institute of Health Sciences, University of Leeds, Leeds, United Kingdom; ^7^Ministry of Agriculture, Lagos, Nigeria; ^8^The Directorate of Planning, Research and Statistics, Nigeria Centre for Disease Control and Prevention, Abuja, Nigeria; ^9^Department of Crop and Horticultural Sciences, University of Ibadan, Ibadan, Nigeria

**Keywords:** urbanization, agricultural intensification, zoonoses, One-Health, Nigeria, URBANE

## Introduction

The global increase in the frequency of infectious-disease epidemics has rekindled interest in the role of disruption of the human–animal–environment interface by human activities in propagating disease outbreaks. It is estimated that zoonotic diseases account for >75% of emerging infectious diseases (1). Human modifications to the environment via agriculture, food-handling practices, deforestation, and changes in ecosystems have led to greater exposure of humans to reservoir hosts and resulted in infectious disease outbreaks. These ecological changes have been linked to climate change with impacts on the survival of pathogens, vector-borne, and food/water-borne diseases. Other anthropogenic drivers of emerging zoonoses include increasing urbanization, migration, civil unrest and globalization (2). These human activities are very potent factors that could drive disease emergence and could be coupled with environmental or geographical drivers such as precipitation, temperature, ecoregions, soil types, and altitude.

A zoonosis is an infectious disease that can be transmitted from animal hosts (3). Zoonotic pathogens may be bacterial, viral, parasitic or fungal and represent a major global public health problem perpetuated by the complex interconnectedness of humans, animals and plants in a shared environment (3). Zoonoses include diseases such as brucellosis, leptospirosis, rabies, onchocerciasis, anthrax, yellow fever, Ebola, Lassa fever, salmonellosis, trypanosomiasis, toxoplasmosis, and taeniasis. With intensification of agriculture, humans have encroached on wildlife habitats resulting in imbalances to the ecosystem whilst putting humans and livestock into closer proximity to wildlife and vectors (4). Following a two-day in-country consultative meeting involving experts from the human, animal, and environmental health backgrounds, rabies, Ebola Virus Disease, swine influenza, avian influenza, and anthrax were ranked as the first five priority zoonoses in Nigeria (5). There has been advocacy for the establishment of community-based One-Health programmes in Nigeria to provide comprehensive epidemiological information necessary to identify and respond to zoonoses (6).

With the global human population projected to reach 9.7 billion by 2050, there is an urgent need to expand and intensify agriculture globally to attain Sustainable Development Goal (SDG) 2, which aims to end hunger, achieve food security, and improve nutrition (7). Over half of the world's people now live in cities as people migrate from rural settlements to cities in search of economic empowerment and livelihood. The drive to increase the volume and frequency of food production globally is being impeded by disruptions in farming occasioned by conflicts and migration of internally displaced persons. Globally, intensified peri-urban farming is occurring with advantages of increasing the resilience of food systems, reducing poverty (Sustainable Development Goal-1) and ensuring nutrition security (Sustainable Development Goal-2). This intensified peri-urban farming comes with concerns for greater occurrence of zoonotic diseases in livestock, sanitary issues from cultivation & livestock-keeping and local environmental degradation from use of synthetic agricultural inputs, pollution and deforestation, risks from pesticides residues, antimicrobial resistance and more.

Therefore, intensified agriculture needs to be balanced with promoting sustainable food systems that cause minimal habitat conversion and decrease zoonotic disease outbreaks. This is more compelling for Nigeria which accounts for more than half (53.5%) of the human population (399, 026, 674) in West Africa in 2021 (8). Zoonotic infections that are endemic in Nigeria include Lassa fever, tuberculosis, salmonellosis, trypanosomiasis, toxoplasmosis, taeniasis, and yellow fever.

In a bid to explore the links between farming practices and human health, a 4-year European Union funded Horizon Europe research and innovation project called URBANE commenced in July 2022. URBANE aims to address the challenge of sustainable agriculture for food nutrition security by applying a One-Health approach to tackling issues related to the application and intensification of peri-urban agriculture. One-Health is an integrated, unifying approach that aims to sustainably balance and optimize the health of people, animals and ecosystems while recognizing that the health of these three entities are closely linked and interdependent. URBANE is built around the principles of agroecology, representing a transdisciplinary field that includes all the ecological, socio-economic, technological, and political dimensions of food systems, from production to consumption, or from farm-to-fork. The project focuses on the West Africa sub-region and uses six experimental farms in Nigeria, Ghana, Morocco, Benin, Burkina Faso, and Senegal. The pilot sites have been carefully selected to cover different agroecological zones, climatic and soil conditions, food habits and sustainability challenges.

The URBANE consortium comprises 26 partners from 16 countries united by the common goal of exploring sustainable agroecological practices and Decision Support Systems (DSS) in peri-urban environments to accelerate the transition toward sustainable food systems (9). To this end, six case studies will be established in Nigeria, Morocco, Senegal, Ghana, Benin, and Burkina Faso. URBANE will be working in close collaboration with farmers, building on local knowledge, supported by new technologies and best practices applied in European regions where agroecology is already applied in intensified, market-oriented production fields. New and adapted business models will also be designed for the URBANE case studies, that will act as lighthouse examples of how such business models can be—with suitable local adaptations—informed by local specificities.

We planned to establish the Nigerian case study in the Tropical Savanna and Forest Agroecological Zones of western Nigeria, essentially in peri-urban towns surrounding Lagos Metropolis. The peri-urban areas that surround Lagos support huge volumes of agricultural activities. The focus would be on strengthening agricultural production systems (both crops and livestock) in a peri-urban setting. In a bid to identify potential study sites, collaborators from the Nigeria Center for Disease Control and Prevention (NCDC), the Nigeria Veterinary Research Institute (NVRI), the University of Ibadan (UI), and the Foundation for Healthcare Innovation and Development (FHIND www.fhind.org) teamed up to conduct a survey in Lagos State Nigeria. The goals of this survey were to obtain an in-dept understanding of the agroecological landscape in a peri-urban setting in Nigeria and identify instances of farming practices, environmental/climatic conditions that led to diseases among humans to help define the Nigeria case study. So, by identifying common pests, and diseases in peri-urban farming in Nigeria we would be able to deploy Industry 4.0 technologies including IoT cost-effective solutions for insect pest population monitoring, livestock health monitoring, soil monitoring, and SWINOSTICS devices for monitoring poultry and pigs.

Another goal of the survey was to establish if there was existing collaboration across the three interdependent sectors—animal health, human health and ecosystems. The information from the survey on agroecological resources in this part of Nigeria would guide the implementation of the URBANE project in Nigeria over a 4-year period. There is scanty published data on crop and animal production in peri-urban settings in Nigeria. Beyond the URBANE project, our survey findings would be of interest to a general audience, as it would provide valuable operational data that could be utilized to plan and implement future agricultural initiatives to improve on the existing services.

This survey was conducted in peri-urban areas namely Agege, Badagry, Ojo, Oke Aro and Ikorodu from 12^th^ to 14^th^ November 2022. Lagos State is situated in the low-lying coastal region of South West Nigeria and bounded geographically by latitudes 6°22′-6°42′ N and longitudes 2°42′-4°20′ E. It is the most populous (but smallest) state in Nigeria and is located in the Rainforest Agroecological Zone with a population of 9,113,605 in 2006. Many farmers in Lagos State are engaged in intensive crop and animal production and the largest pig estate in West Africa can be found here.

**Figure 1:**
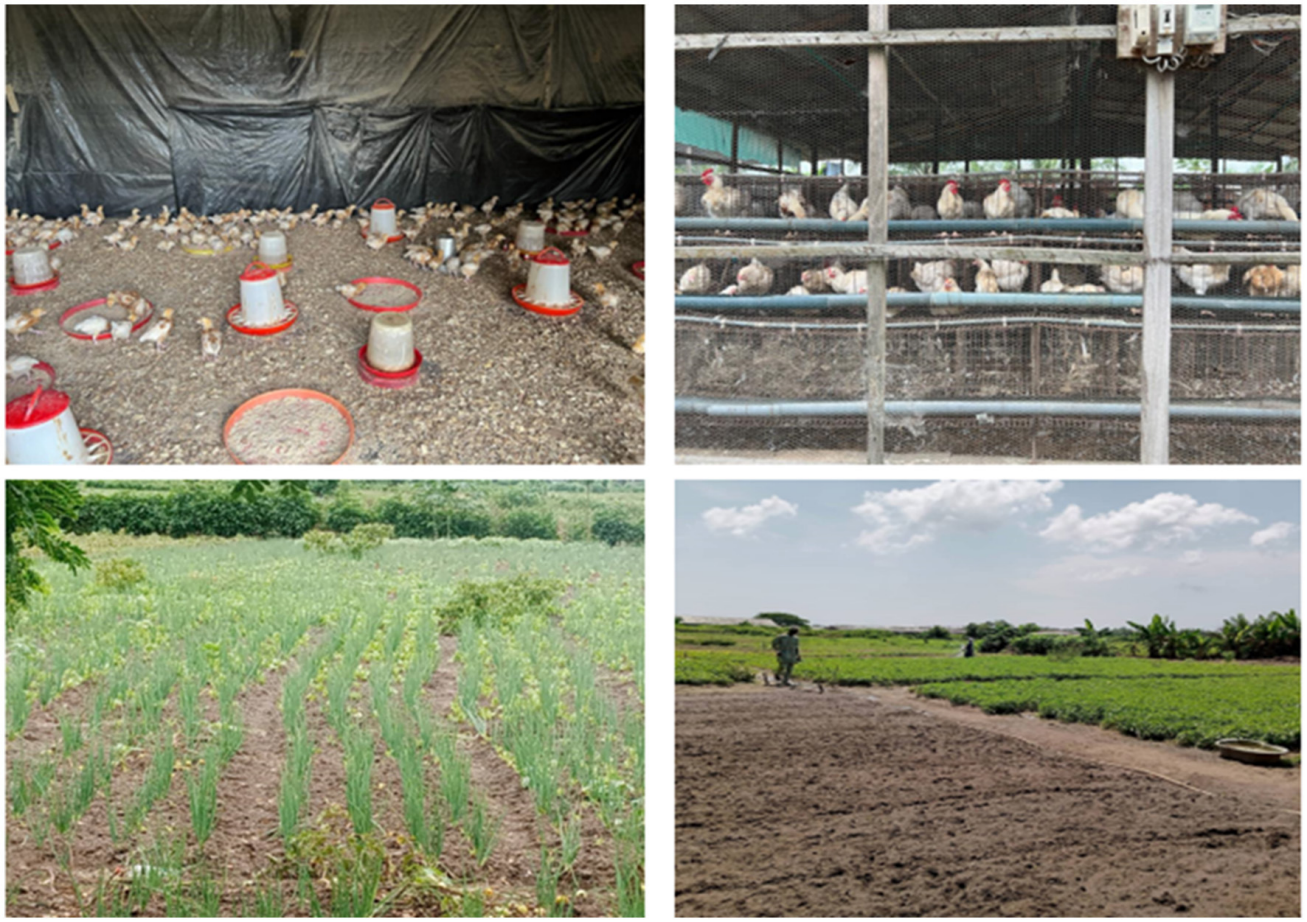
Poultry and crop farming in some of the agricultural enterprises visited in Lagos during the URBANE survey.

Following a pilot survey, a total number of 209 farmers were randomly selected. A questionnaire was developed and administered to crop and animal farmers in Agege (a popular suburb) in Lagos using the Kobo Collect tool (10). A total of 207 responses were obtained from participants comprised of 53 (26%) crop farmers and animal farmers [91 poultry farmers (41%) and 63 pig farmers (30%)] detailing their production practices, challenges, and zoonotic diseases commonly encountered (Figure 1). We were able to categorize the stakeholder groups and collect key information on use of antimicrobials, existing technology used in pest and disease management as well as One-Health related challenges.

## Production practices

### Crop information and production practices

Results from field inspection and responses from 53 crop farmers showed the main crops cultivated in Agege suburbs were mostly Amaranthus cruentus, L. (17.3%), Ocimum gratissimum, L. (17.3%), Corchorus olitorius, L. (14.3%), Telfairia occidentalis, Hook F. (9%), Allium cepa L. (12%), Murraya koenigii, L. (6%) among others. Majority of them (49; 92.5%) cultivated crops in areas less than 3 hectares, 3 (5.7%) utilized between 4–6 hectares for cultivation while only 1 (1.9%) cultivated crops in areas that were more than 6 hectares. Most farmers (38; 60.32%) grew < 5 crops per year, 9 (14.28%) of them grew between 6–10 crops per year while 16 (25.4%) grew more than 10 crops per year.

Arable farming was commonly practiced by 41 (77.4%) of farmers while 12 (22.6%) practiced mixed farming (livestock and arable). The majority of farmers (30; 56.6%) reported selling their produce to retailers, while 21 (41.5%) sold their product to wholesalers with just one (1.9%) farmer sold directly to consumers. A total of 47 (88.7%) of the farmers reportedly applied fertilizers several times per year, with only 1 (1.9%) farmer applying fertilizer once per year and 5 (9.4%) of them admitting to never applying fertilizer on their farms. However, only 27 (13%) of the farmers reported testing their soil to determine its constituents. The most commonly used fertilizer in Nigeria include urea, nitrogen-phosphorus-potassium (NPK) and superphosphate (SSP).

### Livestock information and production practices

A total of 63 (41%) of the animal farmers surveyed were involved in pig production while 91 (59%) were involved in poultry production. Twenty-eight (31%) of the poultry farmers had < 500 birds, 20 (22%) had between 501–1,000 birds, 18 (20%) had between 1,001–1,500 birds, 9(10%) had 1,501–2,000 birds and 16 (17%) had more than 2000 birds. In the pig farms surveyed, 2 (3%) of pig farmers had 201–300 pigs, 2 (3%) of farmers had more than 300 pigs, 10 (16%) had between 101–200 pigs, while 49 (78%) had < 100 pigs. Sixty-seven (43.5%) and 47 (30.5%) of the poultry and pig farmers gave probiotics to their animals with 61 (39.6%) and 33 (21.4%) of the poultry and pig farmers arranging monthly animal health checkups for their animals. The survey results also showed that 21 (13.6%) and 7 (4.5%) of the poultry and pig farmers opted for antimicrobials for treating diseases affecting their animals, while 70 (45.5%) and 56 (36.4%) of the poultry and pig farmers used a combination of antibiotics and local herbal remedies. Vaccination was a strategy adopted by 30 (32.6%) of the poultry farmers and 12 (13%) of the pig farmers to keep the animals healthy.

We were able to obtain key information on crops grown, livestock reared, use of probiotics and antimicrobials in these sites. This baseline information was vital in selecting appropriate case study sites in Nigeria. It also helped the URBANE consortium to decide on the type and number of agroecological tools to be deployed to the Nigeria case study site.

## Health issues related to plants and animals

The main insect pests attacking crops included caterpillars, slugs, white flies, snails, aphids and grasshoppers with 193 (92.5%) of farmers using insecticides to control infestations while 16 (7.6%) of farmers used biological agents. A total of 55 (26.4%) of farmers reportedly used monitoring methods for insect pests with 51 (24.5%) using visual inspection, and 4 (1.9%) using specific traps. One hundred and ninety four (93%) of the farmers reportedly monitored insects daily while the rest (15; 7%) monitored insects weekly.

Major production challenges reported by crop farmers were financial/economic (73.6%), lack of support from experts and agricultural authorities (56.6%), and climate change and degradation of the environment (43.3%). Crop farmers did not report instances of farming practices, environmental/climatic conditions that led to diseases among humans.

On the other hand, the health issues identified in pigs were African Swine Fever (ASF) [49 (78%)], foot and mouth rot [8 (13%)], and diarrhea [4 (6%)], while health issues related to poultry were avian influenza [46 (51%)], Newcastle [21 (23%)], chronic respiratory diseases–[CRD; 15(17%)], Salmonella [3 (3%)], coccidiosis [3(3%)], Gumboro [1(1%)], fowl pox [1(1%)], and coryza [1(1%)].

A total of 68 (86.1%) farmers reported that zoonoses occurred at least once a year on their farms. Most animal farmers [42 (45.7%)] performed vaccination or their animals as a mitigation strategy against zoonosis while 27 (29.3%) preferred hospital/health services visits. A total of 14 (6.5%) of farmers reported not taking any specific actions to prevent their animals from zoonoses. Then main reason provided for this inaction was cost (proposed by 182, 87%). No multi-sectoral strategy involving veterinarians and human health experts appeared to be in place to address the threat posed by these zoonoses to the human communities residing close to these farms.

This survey provided very useful information on the key insect pests and zoonoses in these locations and guided the choice and establishment of the case study for URBANE in Nigeria.

## Use of technology in animal production

A total of 83 farmers (53.9%) stated that they used various forms of technology in animal production. These technology solutions included animal health diagnostics devices [1 (1.2%)], internet support tools [18 (21.7%)], smartphones [46 (55.4%)], and sensing devices [1 (1.2%)] among others. Some of the reasons for not utilizing technological tools in animal production included cost, small farm size, complexity of the technology solutions and lack of supporting structures such as electricity for the tools. The survey revealed the level of adoption of technology for agriculture in these locations and highlighted critical barriers to the wholescale adoption of technology by the farmers in these sites. This information aided the project team in deciding on which technological solutions from URBANE would be relevant to the Nigerian context while mitigating some of the challenges identified from the pilot in the formal rollout.

## Prevailing issues and future directions

Our survey in Lagos state revealed that diseases such as tuberculosis, anthrax, and ASF occurred in the pig farms, while salmonellosis, avian influenza and CRD occurred in poultry farms. The utilization of veterinary care for animals was low with very limited adoption of technological tools for animal production. There was little evidence of collaboration across the three interdependent sectors—animal health, human health and ecosystems—to respond to the threat of zoonotic diseases, notwithstanding that Nigeria was the first country in Africa to launch a One-Health strategic plan for a five-year period (2018–2023) to strengthen a multi-sectoral collaboration for health security.

Our survey revealed that cost was a major barrier to the adoption of preventive strategies for zoonotic diseases among the farmers. It is possible that a greater adoption of One-Health approaches to the issue of controlling zoonotic diseases in Nigeria would reduce the burden of dealing with this on the farmers. In the One-Health strategic plan, the zoonotic diseases that have been given priority include rabies, avian influenza, Ebola, swine influenza, anthrax, tuberculosis, African trypanosomiasis, Lassa fever, *Escherichia coli* O157, and brucellosis some of which were reported by the farmers surveyed in Lagos. A joined up way of working involving the Ministry of Agriculture and Rural Development, the Ministry of Environment, the Ministry of Finance is likely to lessen the burden of preventing zoonotic diseases on individual farmers in Nigeria. Such a multisectoral and multidisciplinary approach to disease control will involve surveillance and response, training and research, communication, governance and leadership, and resource mobilization. Although some gains have been recorded in promoting the One-Health agenda in Nigeria, work is required to ensure a joined up way of working that involves key actors in the agricultural and human health and ecosystem sectors. This needs to be balanced with the urgent need to achieve food security in Nigeria and beyond, given the country's current population which is projected to reach 400 million by 2050.

Other issues identified in our survey was the low level of soil testing among the crop farmers and the low use of traps for monitoring insect pests. Soil testing is an integral part of crop cultivation and allows farmers to measure the level of nutrients in their soil, gauge the appropriateness of growing a particular crop in an area, and adjust their fertilizer application accordingly. The URBANE project will be trialing a low-cost near infrared (NIR) AI-enhanced handheld spectrometer tool by SENS which exploits AI-based chemometrics to measure the soil redox potential (Eh, assessing the availability of electrons) and pH (assessing the availability of protons), and assess soil health (2). As part of the URBANE project a low-cost system developed by CyRIC will be deployed based on automated versions of Delta and McPhail traps for monitoring pests of economic importance that cause damage to the most commonly cultivated crops in the case study sites. Such simple, innovative and low-budget approaches may find applicability in resource-limited settings such as Nigeria. The URBANE project holds promise for providing real world data on the feasibility of deploying such technologies in Nigeria.

The URBANE project in Nigeria will focus on identify local increasing productivity, resilience and nutritional quality of agricultural products by attempting to limit external inputs and increased abiotic and biotic stresses. Nigeria could benefit from the novel, cost-effective tools for decision-support in agriculture and use of probiotics that URBANE is promoting. By promoting greater multi-agency collaboration, it is hoped that sustainable agricultural practices will be adopted and sustained in Nigeria.

Food insecurity is on the rise globally with an estimated 349 million people across 79 countries currently facing acute food shortage—this is up from 287 million in 2021 (11). Food systems continue to be impacted by vulnerabilities such as natural hazards, pests, extreme weather conditions and armed conflicts. Achieving food security locally and on a global scale requires concerted One-Health approaches to prevent economic losses to farmers and stop the spread of zoonotic diseases to animals and humans, and preserve ecosystem health. Increased surveillance, provision of diagnostic and therapeutic options, vaccination of animals as well as education are vital to controlling zoonoses (12).

The in-depth information we obtained on crops grown, livestock reared, use of probiotics and antimicrobials, level of adoption of technology for agriculture in the various locations surveyed will be invaluable in selecting appropriate agroecological solutions for use. Agroecology involves development and scaling of diversified farming systems that use managed ecological processes to address fundamental challenges of farming. In URBANE, the focus would be on managing the ecological processes using synbiotics, in combination with the Advanced Technology Genomics Core (ATGC) technique, SENS scanners to control the aggradation of soils as well as IoT cost-effective solutions for insect pest population monitoring, livestock health monitoring, bio-fertilizer management, soil monitoring, locust swarming prevention and weather monitoring. Organizations within the URBANE consortium have previously published articles on the application of probiotic-based multi-components to human, animal and ecosystem health (13, 14). The SWINOSTICS device will be used for monitoring poultry and pigs. The SWINOSTICS mobile device has the capability of simultaneously analyzing four samples to detect six key swine viral pathogens: Swine Influenza Virus (SIV), African Swine Fever Virus (ASFV), Porcine Parvovirus (PPV), Classical Swine Fever Virus (CSFV), Porcine Respiratory and Reproductive Syndrome (PRRSV), and Circovirus 2 (PCV−2) (15–17). The overall focus will be on creating and piloting a model integrating crops-livestock-fisheries production systems.

The deployment of the URBANE solutions will cut across both agriculture, the environment and human health, as the NCDC will be working closely with NVRI and relevant agricultural agencies to monitor for zoonoses in the case study site. Key barriers to the implementation of the URBANE solutions beyond the scope of the project would include reluctance of farming communities to adopt new technologies and cost as identified in our survey. However, training of the farmers on the use of these devices and supportive supervision would be one mitigating measure. The establishment of multi-agency collaboration and public private partnerships may have the benefit of reducing the cost implications of adopting some of these technologies as production could be increased at lower overall costs thus promoting economy of scale. The deployments of these technologies could strengthen monitoring, identification, reporting systems and control of existing and emerging zoonotic diseases in Nigeria and beyond.

As the URBANE project unfolds in Nigeria, there is hope that its agroecological approaches will engender cross-sectoral stakeholder engagement that could ultimately improve the earth's biodiversity and limit the transfer of diseases from animals to humans and vice-versa.

## Data availability statement

The original contributions presented in the study are included in the article/supplementary material, further inquiries can be directed to the corresponding author.

## Ethics statement

Ethical approval for this project was obtained from the National Health Research Ethics Committee of Nigeria (NHREC) with approval number: NHREC/01/01/2007-10/11/2022.

## Author contributions

AOt, OO, CM, EE, BE, II, CO, and CE conceived the study and drafted the study protocol. OO, CM, II, AOm, JI, SE, and KS were involved with the data gathering from the field survey. KS, OO, and II handled the data cleaning and initial analysis. OO, AOt, II, CO, and VA were involved in further data analysis. AOt and CM wrote the first draft. All authors contributed to various sections of the manuscript and read and approved the final version of the manuscript.
